# Albumin-Mediated Size Exclusion Chromatography: The Apparent Molecular Weight of PSMA Radioligands as Novel Parameter to Estimate Their Blood Clearance Kinetics

**DOI:** 10.3390/ph15091161

**Published:** 2022-09-19

**Authors:** Jan-Philip Kunert, Sebastian Fischer, Alexander Wurzer, Hans-Jürgen Wester

**Affiliations:** Chair of Pharmaceutical Radiochemistry, TUM Department of Chemistry, Technical University of Munich (TUM), 85748 Garching, Germany

**Keywords:** albumin, plasma protein binding, blood clearance, pharmacokinetics, size exclusion chromatography, radioligand therapy, prostate cancer, PSMA, rhPSMA

## Abstract

A meticulously adjusted pharmacokinetic profile and especially fine-tuned blood clearance kinetics are key characteristics of therapeutic radiopharmaceuticals. We, therefore, aimed to develop a method that allowed the estimation of blood clearance kinetics in vitro. For this purpose, ^177^Lu-labeled PSMA radioligands were subjected to a SEC column with human serum albumin (HSA) dissolved in a mobile phase. The HSA-mediated retention time of each PSMA ligand generated by this novel ‘albumin-mediated size exclusion chromatography’ (AMSEC) was converted to a ligand-specific apparent molecular weight (MW_app_), and a normalization accounting for unspecific interactions between individual radioligands and the SEC column matrix was applied. The resulting normalized MW_app,norm._ could serve to estimate the blood clearance of renally excreted radioligands by means of their influence on the highly size-selective process of glomerular filtration (GF). Based on the correlation between MW and the glomerular sieving coefficients (GSCs) of a set of plasma proteins, GSC_calc_ values were calculated to assess the relative differences in the expected GF/blood clearance kinetics in vivo and to select lead candidates among the evaluated radioligands. Significant differences in the MW_app,norm._ and GSC_calc_ values, even for stereoisomers, were found, indicating that AMSEC might be a valuable and high-resolution tool for the preclinical selection of therapeutic lead compounds for clinical translation.

## 1. Introduction

The contribution of nuclear medicine to the clinical management of cancer patients has been on a constant rise throughout the last years [1]. In addition to established diagnostic applications guiding clinical decision making, such as positron emission tomography (PET) [2,3] or single-photon emission computed tomography (SPECT) [4,5], targeted radioligand therapy (RLT) has evolved into an important tool in the treatment of oncological diseases and is still growing in its everyday clinical relevance [6,7,8]. Pioneered by [^177^Lu]Lu-DOTA-TATE [9] (Lutathera^®^) and fueled by its regulatory approval for the treatment of neuroendocrine tumors by the FDA in early 2018 [10], intensive preclinical and clinical research toward novel targeted radiotherapeutics for oncologic targets, such as prostate-specific membrane antigen (PSMA) [11,12], human epidermal growth factor receptor 2 (HER2) [13,14], gastrin-releasing peptide receptor (GRPR) [15,16] and cholecystokinin-2-receptor (CCK2R) [17,18], among others, has been undertaken. The growing patient population suffering from prostate cancer and the resulting clinical demand have especially driven the development of PSMA-targeted radioligands [19,20,21]. As a result, [^177^Lu]Lu-PSMA-617 (Pluvicto^TM^) was recently approved as a first-in-class PSMA-targeted radiopharmaceutical for the RLT of metastatic castration-resistant prostate cancer (mCRPC) [22]. Further developments in radiotracer design and the clinical application of PSMA-targeted RLT in earlier stages of prostate cancer are eagerly awaited to increase the therapeutic options for oncologists and the possible benefit for patients in the near future.

In the development of new PSMA radiotherapeutics, various preclinical in vitro parameters are conventionally assessed to identify lead candidates for clinical translation [23]. Among these parameters, PSMA affinity, as well as uptake and internalization in PSMA-positive cells, are determined to analyze the target binding properties of a radioligand, while lipophilicity is usually assessed to avoid hepatobiliary and to foster renal excretion. In this context, extensive work on the modulation of albumin binding has been carried out with the aim to exploit a depot effect for leveraging increased tumor uptake [24,25,26,27].

However, the optimization of the therapeutic efficacy of a radioligand more than anything requires a meticulously adjusted pharmacokinetic profile [28]. Compared to diagnostic tracers, a slightly slower excretion of therapeutic radioligands is needed to support high tumor uptake, yet excretion must occur fast enough to avoid excessive off-target radiation toxicity [29,30,31]. Despite their undeniable value in preclinical development, the aforementioned in vitro parameters are not suitable to assess, predict, or fine-tune such delicate pharmacokinetic requirements. In contrast, a parameter that governs the complex synergy of tumor delivery and excretion from healthy tissue is the blood clearance kinetics of a radioligand. All relevant pharmacokinetic effects between the site of injection and the tumor cell membrane are influenced by and integrally summarized within the blood clearance kinetics of a ligand. The preclinical assessment of this parameter is, therefore, of utmost importance to select suitable lead compounds for clinical translation and to develop next-generation radiopharmaceuticals with improved therapeutic efficacy.

Therapeutic radioligands are generally designed to be excreted via the renal pathway [29,32]. Consequently, clearance from the blood pool predominantly occurs via glomerular filtration (GF) [33]. According to the kidney’s physiological function, GF is highly size-selective and high-molecular-weight proteins (such as human serum albumin, HSA) are almost quantitatively retained in the blood plasma, while low-molecular-weight compounds (e.g., drugs) are readily filtered [34,35,36]. Therefore, the rate of a compound’s GF and, thus, its clearance from the blood pool can be assumed to be highly dependent on its molecular weight (MW). The availability of a compound for GF, however, is influenced by its binding to plasma proteins, as a small molecule complexed to a high-molecular-weight plasma protein (e.g., HSA) evades GF, resulting in a prolonged circulatory half-life. Assuming metabolic stability, the interplay between plasma protein binding and MW-dependent GF can, thus, be considered the key element shaping the blood clearance kinetics of a renally excreted radioligand.

Several methods to determine the plasma protein binding of drugs have been described in the literature, among them ultrafiltration (UF) [37], different chromatographic procedures [38,39,40], spectroscopic methods [41,42] and even in silico studies on molecular docking and molecular dynamics [43,44]. In the context of radiopharmaceuticals, UF upon incubation in fresh human plasma [45,46,47] or in solutions of HSA [48,49], as well as high-performance affinity chromatography (HPAC) with HSA-modified HPLC columns [26,50,51], represent the most-established approaches that have been applied in the development of diverse PSMA-targeted radioligands. Both methods offer attractive features, such as short analysis times and the possibility of screening-like procedures in HPAC or incubation conditions closely mimicking the in vivo situation before analysis via UF. However, the informative value of these methods remains limited to the mere quantification of a drug’s protein-bound fraction [27,45,46,47,50] or its affinity toward a plasma protein, such as HSA [26,48,51]. The actual need in developing optimized therapeutic radioligands, however, lies in the assessment and interpretation of different renal clearance kinetics of albumin-complexed drugs, especially for those drugs that have very similar plasma protein affinities, plasma protein bindings, and molecular weights, such as PSMA ligands. As the mere ratio of protein binding or the plasma protein affinity of a drug determined by UF or HPAC are insufficient to draw conclusions on their respective quantitative effect on the drug’s renal clearance kinetics, these established methods are not suitable to meet the aforementioned need.

**Figure 1:**
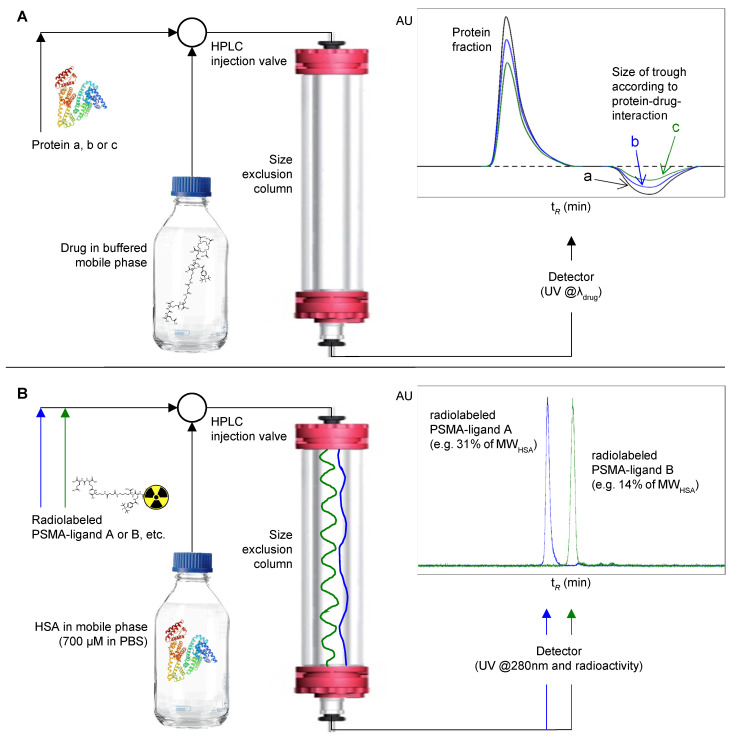
(**A**) Experimental setup of the Hummel–Dreyer method. This method was introduced 1962 to investigate the binding of a drug to proteins. For this purpose, a protein is injected onto a size exclusion column equilibrated with a buffered solution of a drug. After injection, drug molecules from the mobile phase are complexed to the injected protein until equilibrium is reached. The troughs (a, b, c) observed at the cut-off volume in the UV elution profile correspond to the different amounts of drug complexed by the different injected protein samples, thus representing a depleted concentration of the drug at the low-molecular-weight fraction, while the protein fraction shows a stronger signal (protein and complexed drug). (**B**) Experimental setup of the newly developed albumin-mediated size exclusion chromatography (AMSEC). Radiolabeled PSMA ligands are injected onto a size exclusion column equilibrated with a buffered solution of human serum albumin (HSA, physiological concentration of ~700 µM in PBS; pH 7.4). Throughout the chromatographic run, the radioligand binds to and dissociates from HSA in a transient manner. The observed retention time of a radioligand is the result of the mean time the ligand is complexed by HSA, which in turn depends on the strength of the drug–albumin interaction. Thus, an apparent molecular weight (MW_app_) higher than its actual molecular weight (MW) but below the MW of HSA can be assigned to each radioligand.

With respect to these considerations, we aim to develop a method based on size exclusion chromatography (SEC) that not only allows to merely determine the binding of radioligands to HSA but also to possibly deduce conclusions on radioligand blood clearance kinetics. The general feasibility of assessing protein–drug interactions using SEC has been exemplified in the literature by the Hummel–Dreyer method [52]. In this method, a protein is injected onto a size exclusion column equilibrated with a buffered solution of a drug. The depletion of drug concentration caused by protein–drug complexation generates a trough in the UV elution profile observed at the cut-off volume of the chromatogram. Based on the size of the trough, the extent of drug binding to the protein can be determined (Figure 1A). Inspired by the Hummel–Dreyer method and with the aim to account for specific requirements when evaluating radioligands, we developed a novel methodology named albumin-mediated size exclusion chromatography (AMSEC). When compared to the Hummel–Dreyer method, AMSEC works in a reversed fashion: a radioligand is injected onto a size exclusion column equilibrated with a buffered solution of HSA to assess the binding interaction between HSA and the radioligand (Figure 1B).

The aim of this study is to present the development of AMSEC as a novel approach to estimate blood clearance kinetics, to show the method’s basic feasibility as exemplified by the evaluation of ^177^Lu-labeled PSMA radioligands, and to discuss the potential contribution of AMSEC in the future preclinical development of therapeutic radioligands with optimized pharmacokinetics for RLT.

## 2. Results and Discussion

Several considerations of physiological, methodological, and practical nature guided the conceptualization of the AMSEC method. Because of the aforementioned impact of MW on glomerular sieving [34,35,36], we wanted to create an experimental setting capable of producing MW-related data. Therefore, size exclusion chromatography was chosen as the basic technique to assess the interaction between ^177^Lu-labeled PSMA radioligands and HSA. The latter protein represents one of several human plasma proteins that play an important role in the binding of endo- and exogenous ligands [53,54,55]. As HSA is by far the most abundant human plasma protein [56] and its role and function have been intensively studied [54,56,57], we identified this particular protein as the best-suited candidate to evaluate this new method. In contrast to the Hummel–Dreyer method, we decided to apply HSA in the mobile phase rather than to inject a probe of the protein [52]. This holds the distinct advantage that a variety of different PSMA radioligands can be analyzed in a screening-like fashion using the same protein-containing mobile phase and sensitive radioactivity detection for data acquisition (Figure 1B). Furthermore, the mobile phase was composed of HSA at a physiological concentration (700 µm [53]) in phosphate-buffered saline (PBS; pH 7.4), mimicking physiological pH and HSA concentration in blood. Thus, upon the injection of a PSMA radioligand into the HPLC system, HSA and the radioligand interact in solution state and in a flowing, blood-like environment complying with fundamental aspects of the actual in vivo situation.

Despite these thoughtful considerations in the conceptual design of AMSEC, it was initially unclear how the resulting elution profiles would look like and whether valid and quantitative interpretation in terms of the albumin binding and blood clearance of PSMA radioligands would be feasible. The SEC approach provides the necessary conditions for HSA-complexed radioligands and uncomplexed radioligands to behave differently according to the species’ MWs. However, whether single or multiple radiopeaks (e.g., HSA-complexed or uncomplexed radioligands) and, potentially, tailing or fronting of peaks would be observed could not be judged with certainty in advance, as these features of an elution profile are inherently influenced by the binding kinetics and, especially, the lifespan of an HSA–radioligand complex. Dissociation constants (K_d_) in the micromolar range were reported for lipophilic, halogen-, and alkyl-substituted aromatic HSA-binding motifs [58,59]. Theoretically, lifespans of a few milliseconds up to several minutes could arise from these affinities, depending on the rate of dissociation (k_off_) [60]. The radiohybrid (rh) PSMA ligands and further PSMA-binding model compounds (MCs) evaluated in this study contain a lipophilic silicon-fluoride acceptor (SiFA) or a positively charged SiFA (SiFA*lin*) moiety as an albumin-binding motif [61,62,63]. As neither the K_d_ nor the lifespan of HSA complexes with SiFA- or SiFA*lin*-bearing rhPSMA ligands have been investigated so far, our expectations toward the outcome of the first AMSEC experiments were unprejudiced.

**Figure 2:**
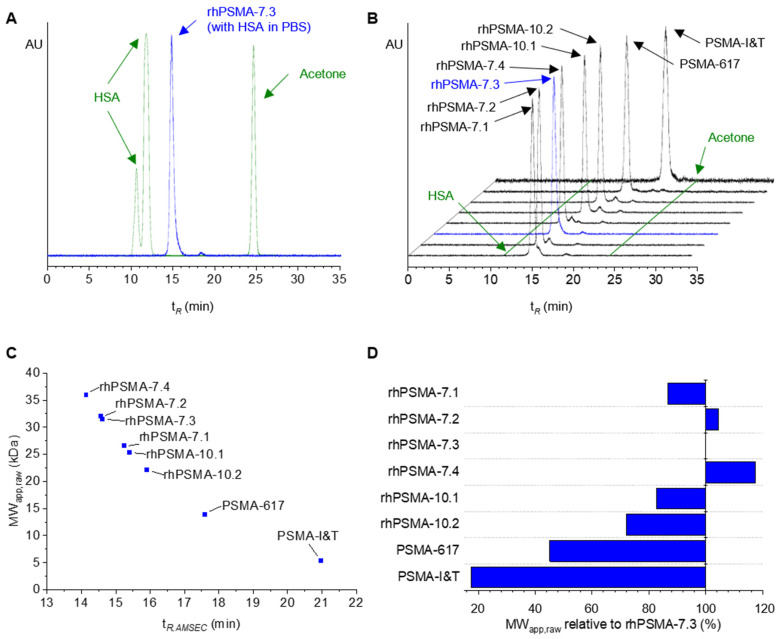
(**A**) Elution profile of rhPSMA-7.3 (blue) in AMSEC experiments (mobile phase of 700 µM HSA in PBS). Elution in the cut-off volume of the column similar to acetone would be expected due to the actual MW of rhPSMA-7.3. Transient binding of rhPSMA-7.3 to HSA during the chromatographic procedure leads to an albumin-mediated reduction in the retention time (t*_R,AMSEC_*) according to the ligand’s HSA-binding strength. The UV peak of HSA indicates the shortest theoretical retention time obtainable for t*_R,AMSEC_* (in the case of continuous HSA-binding, the dominant peak corresponds to the monomeric mass of HSA, and the respective retention time of 11.791 min was used for all calculations). (**B**) Elution profiles of rhPSMA-7.3 (blue line) and rhPSMA-7.1, -7.2, -7.4, rhPSMA-10.1, rhPSMA-10.2, PSMA-617, and PSMA-I&T (black lines) in AMSEC experiments. Retention times of HSA and acetone as references are indicated as green lines. (**C**) t*_R,AMSEC_* of radioligands plotted against the corresponding raw apparent molecular weight (MW_app,raw_). t*_R,AMSEC_* of 14.1–21.0 min led to MW_app,raw_ between 36.0–5.4 kDa. (**D**) MW_app,raw_ of all ligands in percentage of the MW_app,raw_ of rhPSMA-7.3 (100%). Observed MW_app,raw_ ranged from approximately 18% for PSMA-I&T to almost 120% for rhPSMA-7.4.

To our delightful surprise, the injection of ^177^Lu-labeled rhPSMA-7.3 onto the SEC column equilibrated with 700 µm HSA in PBS resulted in a sharp and single radiopeak (Figure 2A). This observation indicated fast binding kinetics (k_on_/k_off_) between HSA and the radioligand. Therefore, within the AMSEC experiment, the transient HSA–radioligand complex might be regarded as a single species. Interestingly, the observed retention time of rhPSMA-7.3 (t*_R,AMSEC_*, 14.722 min) was closer to the retention time of HSA (t*_R,HSA_*, 11.791 min) than to the retention time of acetone (t*_R,acetone_*, 24.809 min, corresponding to the cut-off elution volume), both determined in conventional SEC runs without HSA (Figure 2A). Due to its actual MW (~1.6 kDa) beneath the column fractionation range (70–3 kDa), rhPSMA-7.3 should be eluted in the cut-off volume (like acetone) in the absence of HSA.

We, therefore, concluded that the reduced retention time of rhPSMA-7.3 observed in the AMSEC experiment (t*_R,AMSEC_*) arises from the transient binding of rhPSMA-7.3 to HSA during the passage through the column bed. Whenever rhPSMA-7.3 is complexed to HSA, passage through the column bed occurs rapidly according to the high MW of the HSA–radioligand complex (MW_HSA_ = 66.5 kDa [56]), whereas slow elution according to the ligands’ low actual MW occurs whenever uncomplexed. Consequently, the albumin-mediated retention time t*_R,AMSEC_* observed in the AMSEC run is governed by a ligand’s binding interaction with HSA.

In analogy to the results obtained for rhPSMA-7.3, evaluation of the remaining three isomers of rhPSMA-7, as well as rhPSMA-10.1, rhPSMA-10.2, PSMA-617, and PSMA-I&T, also revealed sharp, single radiopeaks and an individual t*_R,AMSEC_* for each radioligand (Figure 2B). These findings are of particular interest as, by means of column calibration, an apparent molecular weight (MW_app_) can be calculated from the ligand-specific t*_R,AMSEC_*. Such a MW_app_ is higher than the radioligand’s actual, physical MW and arises from transient binding to HSA. With respect to GF, the MW_app_ might be understood as a “biologically effective” MW of the radioligand that describes the relation between albumin binding and the reduced rate of GF, which is commonly observed for albumin-bound drugs. We, thus, hypothesized that, in terms of GF in vivo, the radioligands might pharmacokinetically behave according to their MW_app_ rather than their actual MW. Because of the quantitative correlation between MW and glomerular sieving coefficients (GSCs) [64], the MW_app_ determined by AMSEC could be used to estimate the GF and, thus, the blood clearance of PSMA radioligands.

Within this study, MW_app_ determined directly from experimentally acquired retention times t*_R,AMSEC_* (corrected according to Section 3.3) are referred to as raw apparent MW (MW_app,raw_, Figure 2C,D and Table 1).

As shown in Figure 2C and Table 1, MW_app,raw_ ranging from 5.4 kDa for PSMA-I&T to 36.0 kDa for rhPSMA-7.4 were determined. These values corresponded to a wide range, between 18% and nearly 120% of the value determined for rhPSMA-7.3 (Figure 2D). The latter radioligand served as an internal standard and was assessed whenever AMSEC studies were carried out (n = 14). The resulting mean value for MW_app,raw_ of 30.6 kDa and the low standard deviation of 0.5 kDa showed the high reproducibility of the method. Bearing in mind that the analyzed rhPSMA isomers are structurally highly similar, it constituted a stunning finding that the MW_app,raw_ of, e.g., rhPSMA-7.1 and rhPSMA-7.2, diverged by 20%, even though both isomers structurally differ in only a single stereocenter (chemical structures of ^177^Lu-labeled rhPSMA compounds, PSMA-617, and PSMA-I&T are provided in the Supplementary Materials, Figures S1–S8). This impressive resolution of AMSEC allowing differentiation even between stereoisomers provides an important basis for a possible contribution to the preclinical selection of promising PSMA radioligands.

However, if we critically assess the validity of the determined MW_app,raw_, it becomes apparent that the interpretation of the results presented so far is based on the following hypothesis: the comparability of MW_app,raw_ between different radioligands is only given if the retention times on the SEC column in the absence of HSA are identical for all the radioligands. In this case, any differences observed in t*_R,AMSEC_* among the radioligands would exclusively arise from the ligand-specific interaction with HSA. This uniform retention time in the absence of HSA is furthermore expected to correspond to the cut-off volume, as indicated by the retention time of acetone (t*_R,acetone_*), as the actual MWs of all the radioligands (~1.6 kDa) lies below the column fractionation range (70–3 kDa). In this case, any excess of determined MW_app,raw_ over the actual MW of a radioligand would solely arise from its interaction with HSA in the AMSEC experiment, and the absolute values of MW_app,raw_ would, thus, be precise and meaningful in relation to GF.

**Figure 3:**
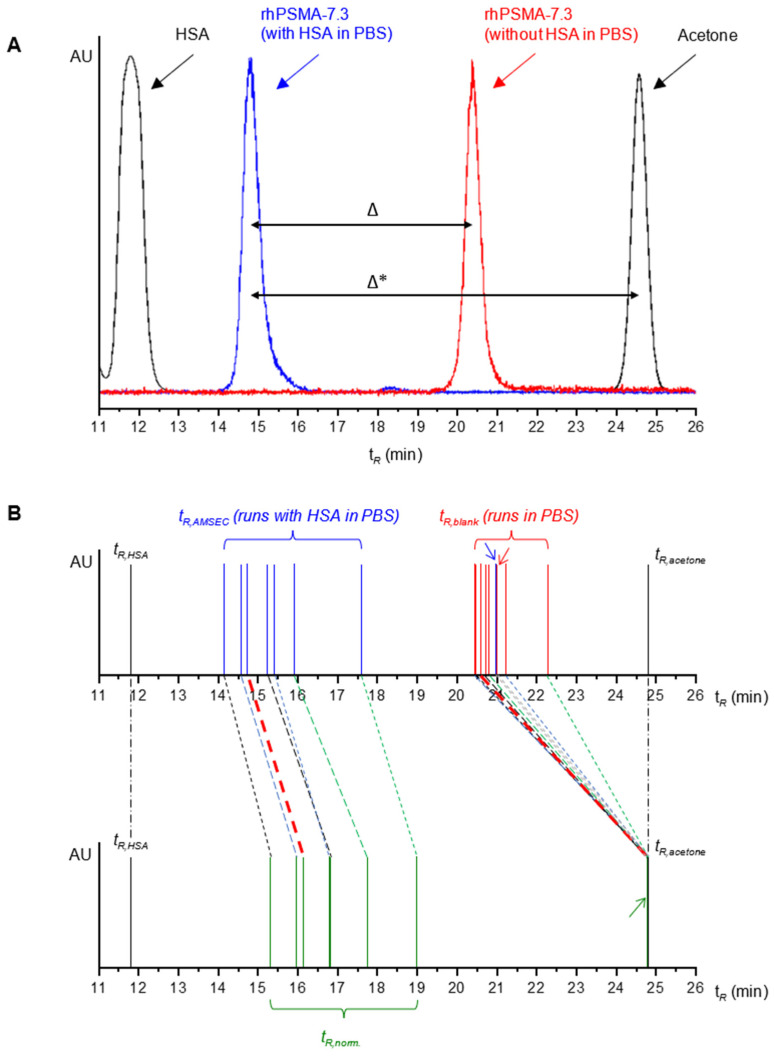
(**A**) The elution profile of the blank run (red) of rhPSMA-7.3 (in PBS without HSA) showed a significantly shorter retention time (t*_R_*) than for acetone, probably due to unspecific electrostatic interactions of the radioligand with the column matrix. The actual albumin-mediated reduction in t*_R_* (Δ) was, therefore, smaller than initially expected (Δ*). (**B**) Graphical depiction of normalization of t*_R,AMSEC_* that was carried out to correct for unspecific interaction with the column matrix. Within the t*_R_* window between HSA (t*_R,HSA_*, 100% HSA binding) and acetone (t*_R,acetone_*, 0% HSA binding), normalized t*_R_* (t*_R,norm._*) were obtained by the proportional dilatation of t*_R,AMSEC_* (ligand-specific HSA binding) and t*_R,blank_*(0% HSA binding) to the extent that all t*_R,blank_* values became equal to t*_R,acetone_*. For the purpose of better readability, the blue, red, and green arrows indicate the t*_R,AMSEC_*, t*_R,blank_*, and t*_R,norm._* of PSMA-I&T, respectively.

To validate this hypothesis, blank runs (PBS pH 7.4 as mobile phase) of the radioligands on the SEC column were performed. The resulting retention times were termed t*_R,blank_* and represent the ligand-specific retention time that would correspond to 0% HSA binding in the AMSEC experiment. Interestingly, a first finding was that rhPSMA-7.3 showed a t*_R,blank_* significantly shorter than t*_R,acetone_*, mathematically corresponding to a MW of 6.0 kDa (Figure 3A). We concluded that unspecific electrostatic interactions between the highly negatively charged radioligand and the agarose/dextran-based matrix of the superdex column are present and lead to an elution prior to the expected cut-off volume [65,66]. Consequently, apart from the radioligand’s binding to HSA in the AMSEC run, the unspecific interactions with the column matrix contribute to the determined MW_app,raw_, thus hampering its informative value. In a similar fashion, all other evaluated radioligands also showed shorter t*_R,blank_* than t*_R,acetone_* values (see Table S1). Furthermore, slightly different t*_R,blank_* between 20.4 min and 22.3 min were observed for the evaluated radioligands (see Table S1). Hence, slightly different windows of retention times represented the whole spectrum, between 100% (t*_R,HSA_*) and 0% HSA binding (individual t*_R,blank_*), for each radioligand, thus compromising the quantitative comparability of the obtained MW_app,raw_. To address these limitations, a normalization of the experimentally obtained t*_R,AMSEC_* was required that would account for ligand-specific interactions with the column matrix and, furthermore, provide a common window of retention times representing 100% to 0% HSA binding that is valid for the determination of the MW_app_ values of all the radioligands.

Such normalization was found to be feasible by means of mathematical transformations that require a radioligand’s retention time in the AMSEC run (t*_R,AMSEC_*) and its retention time in the blank run (t*_R,blank_*), as well as the retention times of HSA (t*_R,HSA_*) and acetone (t*_R,acetone_*) in conventional SEC runs in PBS (pH 7.4). The approach and mathematical implementation are described as follows.

During the AMSEC run, the radioligand is present on the column either in an HSA-complexed or a free, uncomplexed state. Accordingly, at every timepoint, the radioligand moves across the column bed either with the elution velocity of HSA or with the elution velocity of the unbound, free radioligand. As these elution velocities refer to the same column dimensions and are reciprocally proportional to the retention times, the experimental unit of retention time can be used to describe the following correlations.

The retention time of a radioligand in the AMSEC run (t*_R,AMSEC_*) can be expressed as the following linear combination:

(1) $$t_{R,AMSEC}=k\;\cdot \;t_{R,HSA}+\left(1-k\right)\;\cdot \;t_{R,blank},$$

where the retention factor *k* ∈ [0;1] is the fraction of time the radioligand is complexed to HSA during the AMSEC run. Solving Equation (1) for the retention factor *k* gives the following correlation:

(2) $$k=\frac{\left|t_{R,AMSEC}-t_{R,blank}\right|}{\left|t_{R,HSA}-t_{R,blank}\right|}.$$

The term $\left|t_{R,AMSEC}-t_{R,blank}\right|$ in the numerator of Equation (2) represents the shift of the drug´s radiopeak that is observed in the AMSEC run by HSA complexation compared to the blank run. The bigger the shift, the stronger the interaction (and the extent of binding) between a radioligand and HSA. The term $\left|t_{R,HSA}-t_{R,blank}\right|$ in the denominator describes the window of retention times between 100% (t*_R,HSA_*) and 0% HSA binding (t*_R,blank_*) for such shifts. Theoretically, the radiopeak of a particular radioligand in the AMSEC run is observed within this window. Thus, the denominator in Equation (2) describes the base reference to comparably quantify the extent of binding expressed by the numerator. According to these correlations, a radioligand with a retention factor of, e.g., *k* = 0.5, would exhibit a t*_R,AMSEC_* exactly in the middle between t*_R,HSA_* and t*_R,blank_*. The retention factor *k* is dimensionless and independent of ligand-specific interactions with the column matrix. It is, thus, a quantitative and ligand-specific parameter for HSA binding that allows for the comparison of the HSA binding between different compounds.

In Equation (2) the ligand-specific retention time t*_R,blank_* represents 0% HSA binding. As stated earlier, a common point of reference equaling 0% HSA binding for all radioligands is required to determine MWs that are quantitatively comparable. Acetone was chosen as this reference, as this small molecule does not exhibit any unspecific interactions with the agarose/dextran-matrix of superdex columns and is eluted within the cut-off volume of the column. The latter was theoretically also expected for the radioligands evaluated herein, as their actual MW lies beneath the fractionation range of the column. The implementation of t*_R,acetone_* into Equation (2) gives the following equation:

(3) $$k=\frac{\left|t_{R,norm.}-t_{R,acetone}\right|}{\left|t_{R,HSA}-t_{R,acetone}\right|},$$

with the normalized retention time t*_R,norm._* replacing t*_R,AMSEC_*. The normalized retention time t*_R,norm._* is the virtual retention time of a radioligand that, within the newly defined window of 100% (t*_R,HSA_*) to 0% HSA binding (t*_R,acetone_*), results in the same retention factor *k* determined in Equation (2). According to Equation (3), t*_R,norm._* is defined as follows:

(4) $$t_{R,norm.}=k\cdot t_{R,HSA}+\left(1-k\right)\cdot t_{R,acetone}.$$

Combining Equations (2) and (4), t*_R,norm._* can finally be determined from solely experimental input factors as follows:

(5) $$t_{R,norm.}=\frac{\left|t_{R,AMSEC}-t_{R,blank}\right|}{\left|t_{R,HSA}-t_{R,blank}\right|}\cdot t_{R,HSA}+\left(1-\frac{\left|t_{R,AMSEC}-t_{R,blank}\right|}{\left|t_{R,HSA}-t_{R,blank}\right|}\right)\cdot t_{R,acetone}.$$

Consistent with the comparability of *k*, t*_R,norm._* is also comparable among different radioligands and can be used to calculate a normalized apparent MW (MW_app,norm._) by means of column calibration. The corresponding data of the aforementioned rhPSMA compounds, PSMA-617, PSMA-I&T, and ten further model compounds (MC-1 to MC-10) are presented in Table 2 (t*_R,AMSEC_* and MW_app,raw_ of MC-1 to MC-10 are provided in Table S2).

In addition to the mathematical derivation, the presented normalization can also be understood in a geometrical way when the peaks of HSA (t*_R,HSA_*, 100% binding), the radiopeak in the AMSEC run (t*_R,AMSEC_*, ligand-specific binding to HSA), and the radiopeak in the blank run (t*_R,blank_*, no HSA in mobile phase, thus 0% HSA-specific binding) are thought of as one peak profile. The described mathematical transformations correspond to a proportional dilatation of that peak profile to the extent that the shifted t*_R,blank_* values become equal to t*_R,acetone_*, the new uniform point of reference for 0% HSA binding, while t*_R,HSA_* is kept unaltered (see Figure 3B).

As the normalization provides both a correction of the ligand-specific interactions with the column matrix and a common window of retention times between 100% and 0% HSA binding, the obtained MW_app,norm._ are comparable among the different radioligands and the increase in MW_app,norm._ compared to the actual MW of a radioligand is solely HSA-mediated. We, therefore, consider MW_app,norm._ to be the valid and informative parameter that can be obtained by applying AMSEC in the preclinical development of PSMA radioligands.

Only the above-described normalization of the AMSEC data on the binding of radioligands to HSA provides MW-related data with the unique advantage that conclusions related to the MW-dependent physiological process of GF might be drawn, allowing for the estimation of the relative blood clearance kinetics of different radioligands.

**Figure 4:**
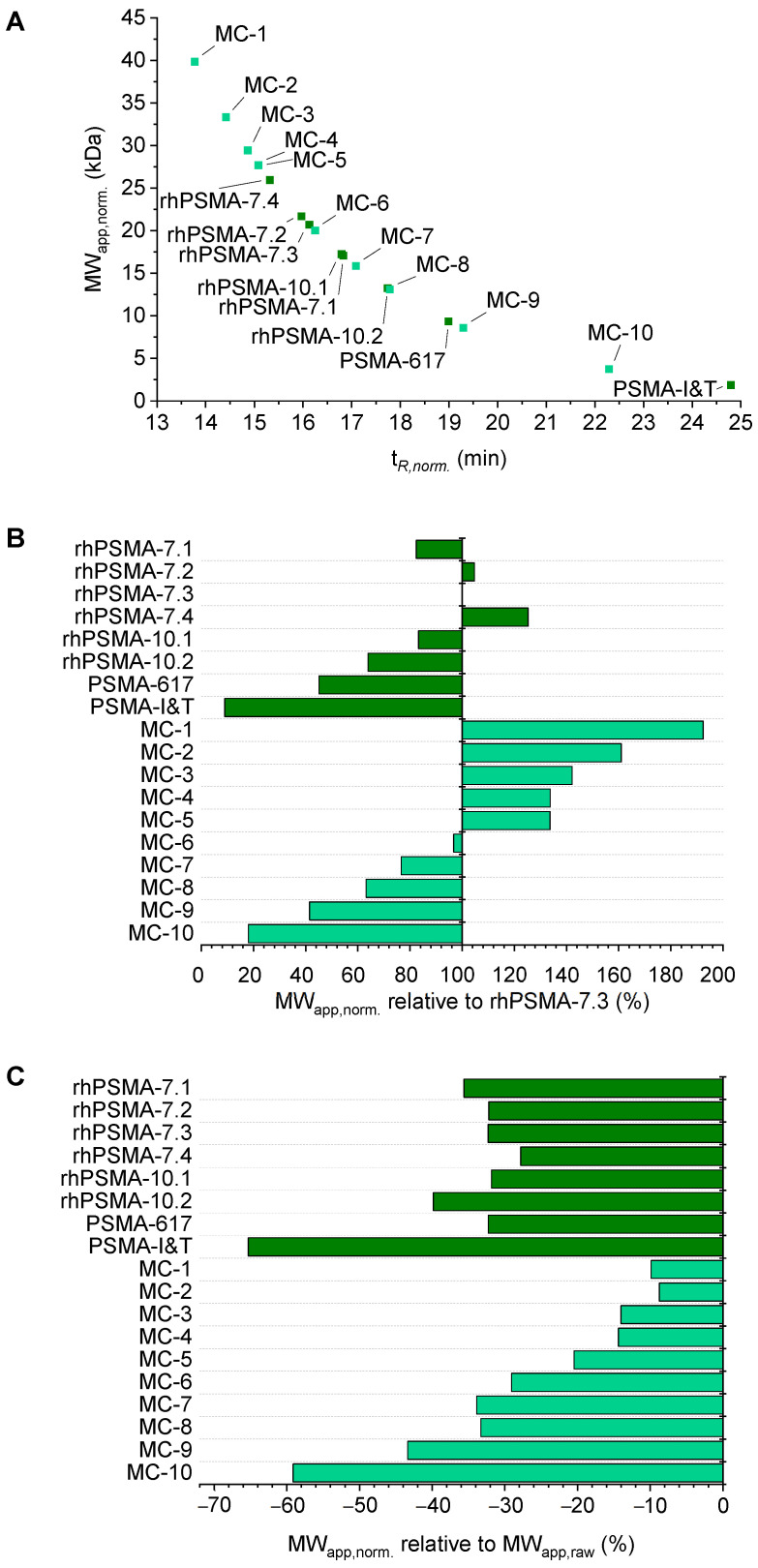
(**A**) Correlation between t*_R,norm._* of radioligands and the corresponding normalized apparent molecular weight (MW_app,norm._). t*_R,norm._* of 13.8–24.8 min corresponded to MW_app,norm._ between 39.9–1.9 kDa. (**B**) MW_app,norm._ of all ligands in percentage of the MW_app,norm._ of rhPSMA-7.3 (100%). Observed MW_app,norm._ ranged from approximately 10% to almost 190% of the MW_app,norm._ of rhPSMA-7.3. (**C**) Relative reduction in MW_app,norm._ compared to their corresponding MW_app,raw_. The extent of the effect of the normalization was dependent on the molecular structure of the radioligand and ranged between −10% and −65% for the compounds presented in this work.

Apart from rhPSMA compounds, PSMA-617, and PSMA-I&T, ten further model compounds (MC-1 to MC-10, including SiFA- and SiFA*lin*-bearing PSMA ligands) were evaluated using AMSEC to show the broad range of MW_app,norm._ values that could be obtained for structurally different compounds. MW_app,norm._ values from 1.9 kDa to 39.9 kDa were observed, which corresponds to a range of 9% to 192% of the MW_app,norm._ determined for rhPSMA-7.3 (Figure 4A,B). As already stated in the context of MW_app,raw_, distinct differences even between stereoisomers were also found for MW_app,norm._. For example, a 27% higher value was found for rhPSMA-7.2 than for rhPSMA-7.1 (21.7 vs. 17.1 kDa, respectively), and even a 30% higher value was found for rhPSMA-10.1 than for rhPSMA-10.2 (17.2 vs. 13.2 kDa, respectively). These findings suggest an impressive resolution of AMSEC, which might be of considerable value, especially for the diligent structure optimization of a therapeutic lead compound.

Within the set of acquired data, however, the single lowest MW_app,norm._ of 1.9 kDa determined for PSMA-I&T needs to be treated with caution. In contrast to all the other radioligands, the evaluation of PSMA-I&T yielded nearly identical t*_R,AMSEC_* and t*_R,blank_* (20.983 min and 20.995 min, respectively) leading to a disproportionately low retention factor *k* of only 0.0013. Consequently, a high t*_R,norm._* of 24.792 min comparable to t*_R,acetone_* was determined, resulting in a suspiciously low MW_app,norm._ for PSMA-I&T. This apparent lack of interaction between HSA and PSMA-I&T seems unreliable, as an albumin-binding capacity was reported for PSMA-I&T [67]. Apart from electrostatic interactions, the delayed elution of analytes bearing (multiple) aromatic residues caused by attractive hydrophobic interactions with crosslinked polysaccharide-based SEC gels (e.g., superdex and sephadex) has been reported by several groups [68,69,70]. PSMA-I&T bears a patch of two hydrophobic aromatic amino acids, namely phenylalanine and halogenated 3-iodo-tyrosine. We hypothesized that this unique structural feature might lead to a complex interplay of electrostatic and hydrophobic interactions for this particular derivative, possibly leading to an underestimation of its MW_app,raw_ and MW_app,norm._. Thus, AMSEC data for PSMA-I&T might presumably be compromised by these hydrophobic interactions and, therefore, valid comparability with the data obtained for the remaining radioligands might not be given for PSMA-I&T.

Since the normalization eliminated contributions to MW_app,raw_ caused by ligand-specific interactions, presumably electrostatic interactions with the column matrix, it was not surprising that values for MW_app,norm._ were smaller than the respective MW_app,raw_ values. It is noteworthy, however, that the relative effect of the normalization on MW_app,raw_ was very different for the radioligands. As shown in Figure 4C, normalization resulted in MW_app,norm._ values reduced by only about 10% (MC-1 and MC-2) to around 60% (PSMA-I&T and MC-10) of the original MW_app,raw_. Among the rhPSMA compounds and PSMA-617, the influence of the normalization was comparable (approximately 30% to 40% reduction). There seems to be a trend of diminished influence of the normalization for radioligands with high MW_app,raw_ values. This phenomenon can be explained by the fact that the aforementioned electrostatic interactions between radioligands and the column matrix only occur when a radioligand is not complexed to HSA. As radioligands with high HSA binding are mainly complexed to the protein (reflected in high retention factors *k*), the influence of the electrostatic interactions on MW_app,raw_ is accordingly smaller and the MW_app,norm._, hence, deviates to a lesser extent from MW_app,raw_ than for radioligands with weaker HSA binding (e.g., significantly lower MW_app,raw_).

**Figure 5:**
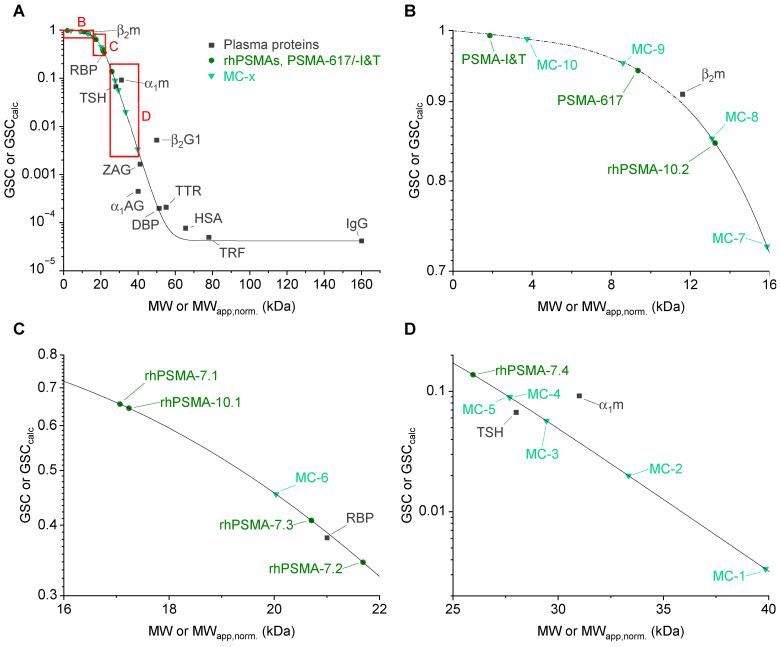
(**A**) Correlation between molecular weights (MWs) of plasma proteins and their glomerular sieving coefficients (GSCs, data from Norden et al. [64]). α1AG: α1-acid glycoprotein; α1m: α1-microglobulin; HSA: human serum albumin; β2G1: β2-glycoprotein I; β2m: β2-microglobulin; DBP: vitamin-D-binding protein; IgG: immunoglobulin G; RBP: retinol-binding protein; TRF: transferrin; TSH: thyroid-stimulating hormone; TTR: transthyretin; ZAG: zinc-α2-globulin. MW_app,norm._ of PSMA radioligands were plotted against their respective calculated GSCs (GSC_calc_). Panels (**B**–**D**) show different zoom-ins on PSMA radioligands, as depicted with red squares in panel (**A**). The extrapolation (dotted line) of the sigmoidal fit of GSC against MW in panel (**B**) was eye-fitted.

Regarding the pharmacokinetically relevant conclusions that might be drawn from the determined MW_app,norm._, it is important to bear in mind that the correlation between MW and GSC and, thus, GF is not linear but shows a sigmoidal curve. Therefore, when comparing the MW_app,norm._ of different radioligands, not only their relative difference but also the absolute values of MW_app,norm._ are of importance. To gain a more intuitive understanding of how a certain MW_app,norm._ might influence blood clearance by means of GF, we used a sigmoidal fit of the MWs and glomerular sieving coefficients (GSCs) of 12 human plasma proteins (Figure 5, data reported by Norden et al. [64]) to determine calculated GSCs (GSC_calc_) from MW_app,norm._. These GSC_calc_ determined for all the PSMA radioligands (Table 2, Figure 5) inherently account for the exponential nature of the relationship between their albumin-mediated MW_app,norm._ and MW-dependent GF and could, therefore, be a useful parameter to estimate differences in blood clearance by means of GF. The GSC is defined as the ratio of a compound’s concentration in the glomerular filtrate to that in the blood [64]; thus, a GSC of 1 represents unhindered filtration through the capillary membrane in the glomerulus, while a lower GSC corresponds to a restricted filtration and, thus, a prolonged circulatory half-life. Interestingly, the determined GSC_calc_ values for the evaluated PSMA radioligands covered a wide range, from values above 0.95 (PSMA-I&T, MC-9, and MC-10), which correlates to mostly unhindered GF, to values below 0.05 (MC-1 and MC-2), which should be associated with high blood retention. The GSC_calc_ of rhPSMA-compounds were more moderate, yet they varied significantly among each other (0.138 to 0.846), even in the subgroup of the isomers of rhPSMA-7. As an example, a GSC_calc_ of 0.408 was determined for rhPSMA-7.3, while an almost three-fold smaller GSC_calc_ of 0.138 was determined for rhPSMA-7.4. Accordingly, if we assume GF to be the only contribution of renally induced blood clearance, a three-fold decelerated blood clearance should be expected for rhPSMA-7.4 compared to rhPSMA-7.3. This would constitute a dramatic physiological effect that, in its extent, could not be predicted only based on subtle differences in molecular structure and other conventionally determined in vitro parameters. We, therefore, believe that the MW_app,norm_. and GSC_calc_ obtained by AMSEC could be important parameters that, already at the preclinical stage of development, could help in the selection of promising therapeutic radioligands with pharmacokinetic profiles suitable for patient application.

Regarding the current state of the art in the RLT of mCRPC, recently approved ^177^Lu-labeled PSMA-617 is considered the gold standard, and rapid, bi-phasic blood clearance was reported for this radioligand [71]. In the past years, considerable efforts have been made by various groups to develop derivatives of PSMA-617 with improved therapeutic efficacy via the incorporation of a dedicated albumin-binding entity [24,25,26]. Preclinical therapy studies in mice of, e.g., ^177^Lu-labeled EB-PSMA-617 and PSMA-ALB-56, have shown superior therapeutic efficacy compared to PSMA-617 [24,25] and have raised hopes for a similar performance in patients. Lamentably, increased blood retention in patients resulted not only in increased tumor dose but also in disproportionately higher doses to most healthy tissues, among them bone marrow and kidneys, two potentially dose-limiting organs in PSMA-targeted RLT [72,73]. Consequently, the resulting inferior therapeutic efficacy indicates that the blood clearance of these novel radiotherapeutics might already be too slow. An intrapatient dosimetry comparison between ^177^Lu-labeled radioligands rhPSMA-7.3 and PSMA-I&T (an established PSMA ligand for RLT with similar pharmacokinetics as PSMA-617 [74]) showed a 2.4-fold higher tumor dose for rhPSMA-7.3 [75]. However, nearly identically increased doses to kidneys and bone marrow resulted in an overall similar therapeutic efficacy of both radioligands. Once more, these findings could not keep promises made by preclinical therapy studies that had indicated a higher therapeutic efficacy for rhPSMA-7.3 than for PSMA-I&T [76], which once again underlines the so far unmet need of valid preclinical indicators for a pharmacokinetic profile yielding improved therapeutic efficacy in patients.

Considering the aforementioned clinical data, it might be suggested that the sweet spot of albumin binding and, thus, the narrow window of optimal blood clearance for improved therapeutic efficacy may actually lie between rhPSMA-7.3 and PSMA-617. In that light, rhPSMA-10.1 might be an attractive candidate for RLT of mCRPC. In a recent preclinical study in mice, rhPSMA-10.1 revealed fast blood clearance and improved tumor-to-background ratios in a head-to-head comparison with four isomers of rhPSMA-7 and rhPSMA-10.2 [62]. Furthermore, our study determined a MW_app,norm._ of 17.2 kDa and a GSC_calc_ of 0.644 for rhPSMA-10.1. Thus, rhPSMA-10.1 could possibly exploit this interesting window of blood clearance kinetics between rhPSMA-7.3 (MW_app,norm._: 20.7 kDa; GSC_calc_: 0.408) and PSMA-617 (MW_app,norm._: 9.4 kDa; GSC_calc_: 0.942). However, clinical data are needed to verify this assumption, and a currently initiated, integrated phase 1 and 2 study with ^177^Lu-labeled rhPSMA-10.1 (clinicalTrial.gov identifier: NCT05413850) will hopefully shed light on that question.

Regarding the limitations of the newly developed AMSEC method, it needs to be stated that the mobile phase composition did not account for the influence of plasma proteins other than HSA (e.g., alpha-1-acid glycoprotein [55,56]) on the pharmacokinetics of the evaluated radioligands. As discussed earlier, HSA is the most abundant human plasma protein and, next to its dominant role in the binding of exogenous compounds, HSA also meets the practical requirement of commercial availability in decent quantities. While a further refinement of the mobile phase in AMSEC, e.g., by supplementation with other relevant human plasma proteins, exceeds the scope of this study, it might be a worthwhile approach for future research.

The feasibility of a normalization accounting for the unspecific, presumably electrostatic interactions of PSMA ligands with the agarose/dextran-based column matrix was an important finding of the present study. However, the aformentioned inconclusive results obtained for PSMA-I&T presumably caused by hydrophobic interactions that were (among the evaluated radioligands) seemingly unique to this particular compound emphasize the complexibility of possible interactions between peptidic ligands and gel filtration matrices. Therefore, more detailed studies evaluating the interactions between a superdex matrix and peptidic radioligands are necessary to elucidate this explicit case. A broader understanding of the unspecific interactions between radioligands and column resin would, furthermore, help to ascertain whether further series of peptidic radioligands other than PSMA show largely deviating, group- (or better structure-) specific interactions with a superdex matrix. Thus, it remains to be investigated with a variety of other peptidic radioligands to various targets whether comparable HSA binding also results in comparable MW_app,norm._ values and, thus, comparable GSC_calc_ or whether MW_app,norm._ primarily allows only for the differentiation of relative GF rates within a group of structurally highly similar ligands.

Another limitation constitutes the fact that HSA is a biologic product and batches might vary according to the origin of the plasma samples that were used in production. For example, results obtained in this study could not be adequately reproduced with HSA of another supplier. To examine the influences of different batches of HSA on the obtained data, however, exceeds the scope of this study and remains to be investigated.

Finally, in order to generally assess the validity of data generated by AMSEC for the estimation of blood clearance kinetics, clinical data of the evaluated PSMA radioligands in patients are necessary. To date, most of the available data describe the dosimetry and biodistribution of PSMA-617, and data on blood clearance (e.g., time–activity curves) are reported for the latter [71,77,78] and some other PSMA radioligands [72,73,79]. However, applied protocols to determine blood clearance kinetics vary among different studies (e.g., serial blood sampling [71,73,79] vs. image-based approaches [72] and varying or unmentioned parameters for the fitting of time–activity curves [73,77,79]), thus hampering comparability of the reported results. Furthermore, direct inter- or intrapatient comparisons of the blood clearance of PSMA radioligands evaluated herein (e.g., rhPSMA-7.3 vs. PSMA-617 or rhPSMA-7.3 vs. rhPSMA-10.1) are currently lacking. Consequently, even though relative trends, e.g., slower blood clearance of rhPSMA-7.3 compared to PSMA-617, can be deduced from data reported in different comparative studies, the current state of our knowledge does not yet allow for a valid correlation of MW_app,norm._ and GSC_calc_ obtained in our study. However, ongoing research and clinical studies, e.g., on rhPSMA-10.1 (NCT05413850), will provide more data in the future and, thus, will help to validate and interpret parameters such as MW_app,norm._ and GSC_calc_ with more accuracy. Furthermore, AMSEC studies of other PSMA radioligands with reported clinical data could help sharpen our understanding of the actual correlation between MW_app,norm._ and blood clearance kinetics in vivo.

In summary, the newly developed AMSEC method allows for the determination of HSA-mediated MW_app,norm._ values of PSMA radioligands. High reproducibility was observed, and the resolution of differences in MW_app,norm._ values, even among stereoisomeric radioligands, was feasible. The novel parameter of MW_app,norm._ could serve as a valuable tool in the preclinical development of predominantly renally excreted therapeutic PSMA radioligands, as blood clearance by means of GF is a highly MW-dependent physiological process. According to the correlation between MW and glomerular sieving, we thus suggest the determination of GSC_calc_ from MW_app,norm._ as a means to assess different blood clearance kinetics of PSMA radioligands in vitro. However, a broader availability of comparable clinical data on the pharmacokinetics of the radioligands evaluated herein is needed to validate and specify the predictive values of MW_app,norm._ and GSC_calc_.

## 3. Materials and Methods

### 3.1. Instrumentation and Software

A high-performance liquid chromatography (HPLC) gradient system from Shimadzu (Neufahrn, Germany) equipped with LC20-AD gradient pumps and a SIL-20A HT autosampler was used for all chromatographic experiments. Detection of UV signals was carried out using an SPD-20A UV-vis detector, and radioactivity was detected with a HERM LB 500 NaI detector (Berthold Technologies, Bad Wildbad, Germany). Chromatograms were analyzed using LabSolutions software from Shimadzu. The readout of radio thin-layer chromatography (radio-TLC) for quality control of radiolabelings was carried out using a Scan-RAM detector and Laura software (LabLogic Systems, Sheffield, UK). Microsoft excel (Redmond, WA, USA) was used for all calculative evaluations. OriginPro software (v9.7) from OriginLab (Northampton, MA, USA) was used for sigmoidal curve fitting.

### 3.2. Preparation of ^177^Lu-Labeled PSMA Radioligands

The four isomers of uncomplexed rhPSMA-7 (7.1 to 7.4) and the two isomers of uncomplexed rhPSMA-10 (10.1 and 10.2) were synthesized as described earlier [62]. Compounds MC-1 to MC-9 represent PSMA-SiFA compounds, and compound MC-10 represents a PSMA-SiFA*lin* compound. PSMA-617 was supplied by MedChemExpress LLC (Monmouth Junction, NJ, USA), and PSMA-I&T was prepared according to the published procedure [80].

Radiolabeling with no-carrier-added [^177^Lu]LuCl_3_ (specific activity > 3000 GBq/mg at the time of radiolabeling, 740 MBq/mL, 0.04 m HCl, ITM, Garching, Germany) was carried out according to a previously established procedure [62] with molar activities of 10–20 GBq/µmol. Quality control of radiolabelings was performed using either analytical reversed-phase (RP) HPLC or radio-TLC. For analytical RP-HPLC, the HPLC-system described above was equipped with an RP column (MultoKrom 100C18, 150 × 4.6 mm, 5 μm; Multospher 100RP18, 125 × 4.6 mm, 5 μm; CS Chromatographie Service, Langerwehe, Germany), and linear gradients of water (solvent A, + 0.1% TFA, *v*/*v*) and acetonitrile (solvent B, +2% water, +0.1% TFA, *v*/*v*) were applied. Quality control via radio-TLC was performed using 1.0 m NH_4_OAc/DMF (1/1, *v*/*v*) on TLC silica gel 60 F_254_ plates (Merck Millipore, Burlington, VT, USA) or 0.1 m sodium citrate buffer on iTLC-SG chromatography paper (Agilent, Santa Clara, CA, USA). Radioligands were used for experiments if the radio chemical purity (RCP) was > 95%.

All data presented in this work were obtained with the ^177^Lu-labeled species of PSMA ligands. For ease of readability, the lutetium complex is omitted in the designation of all radioligands in running text, figures, and tables (e.g., “rhPSMA-7.3” instead of “[^177^Lu]Lu-rhPSMA-7.3” or “MC-1” instead of “[^177^Lu]Lu-MC-1”).

### 3.3. AMSEC Experiments

All size exclusion chromatographic (SEC) experiments were carried out using the above-mentioned HPLC system equipped with a Superdex 75 Increase 10/300 GL gel filtration size exclusion column (fractionation range 70–3 kDa, GE Healthcare, Uppsala, Sweden). Eluents at room temperature, a constant flow rate of 0.8 mL/min, and an acquisition time of 35 min were generally applied. The column was calibrated as recommended by the manufacturer using a commercially available set of proteins (Gel Filtration LMW Calibration Kit, GE Healthcare, Buckinghamshire, UK). Retention times of calibration proteins and calculated calibration parameters are given in the Supplementary Materials (Table S3). UV signal detection occurred at 280 nm.

All retention times of radio signals were corrected for the offset between the UV-vis detector and radio-detector (Δt = 0.084 min). Throughout the development of the AMSEC method, three gel filtration columns of the same type were used for experiments (most data were acquired using column 1). As calculations were based on absolute empirical retention times that inherently differed slightly from column to column, all retention times determined on column 2 and column 3 were mathematically converted to an equivalent retention time on column 1 using the columns’ calibration parameters. A detailed description of these calculations is provided in the Supplementary Materials. A tabular summary of corrected retention times for all compounds is given in Table S1.

All numerical retention times explicitly given in running text or tables and plotted in diagrams are already corrected for the offset between UV-vis and radio-detector and, if initially determined on columns 2 or 3, converted to the equivalent retention time on column 1 (for detailed information, see Equations (S1) and (S2) and Table S1 in the Supplementary Materials).

#### 3.3.1. Determination of Raw Apparent Molecular Weight (MW_app,raw_)

AMSEC runs were executed with a solution of HSA (Biowest, Nuaillé, France) in PBS (pH 7.4) at physiological concentration (700 µm) as a mobile phase. Upon injection of approximately 1.0 MBq (5–10 µL) of a radioligand, the elution profile was monitored via radioactivity detection, and the retention times of observed radiopeaks (t*_R,AMSEC_*) were determined via semi-automated peak integration. By means of calibration, retention times were translated into a ligand-specific raw apparent molecular weight (MW_app,raw_) as a parameter to assess the extent of HSA binding (for calculation, see Equation (S6) in the Supplementary Materials). rhPSMA-7.3 served as an internal standard during AMSEC studies and was co-evaluated whenever new data were collected to assess the reproducibility of the method (n = 14).

For reference purposes, conventional SEC runs of HSA (100 µL, 3 mg/mL) and acetone (100 µL, 2% in PBS pH 7.4) with PBS (pH 7.4) as a mobile phase were executed. The retention time of HSA (t*_R,HSA_*) corresponded to the maximal MW_app,raw_, theoretically observable in AMSEC runs in the case of continuous binding of a radioligand to HSA (equivalent to 100% HSA binding). The retention time of acetone (t*_R,acetone_*) served as a reference for a ligand with no interaction with HSA in AMSEC runs (equivalent to 0% HSA binding) and an actual physical molecular weight below the column fractionation range (this was the case for all investigated radioligands).

#### 3.3.2. Determination of Normalized Apparent Molecular Weight (MW_app,norm._)

MW_app,raw_ values determined as described in Section 3.3.1. were normalized in order to account for ligand-specific influences on experimentally determined retention times and to establish quantitative comparability of the obtained MW_app,norm._ values. The normalization was based on a SEC run (referred to as blank run) of a radioligand executed in analogy to the AMSEC run but using PBS (pH 7.4) without HSA as a mobile phase. The determined retention time t*_R,blank_*, together with the retention time t*_R,AMSEC_* from the AMSEC run, t*_R,HSA_*, and t*_R,acetone_* determined in Section 3.3.1, were used to calculate a normalized retention time t*_R,norm._* for every radioligand (for formulae and details on the calculation, see Section 2. Results and Discussion). The MW_app,norm._ values were subsequently calculated from t*_R,norm._* by means of column calibration (an exemplary calculation is provided within Equation (S3) in the Supplementary Materials).

### 3.4. Determination of Calculated Glomerular Sieving Coefficients (GSC_calc_)

A sigmoidal fit of molecular weights (MWs, in kDa) and corresponding glomerular sieving coefficients (GSCs) of 12 human plasma proteins (data taken from Norden et al. [64]) was executed using OriginPro software. A dose-response model based on Equation (6) was applied:

(6) $$GSC=A1+\frac{A2-A1}{1+10^{\left(LOGx0-MW\right)\cdot p}}.$$

The values of bottom asymptote A1 and top asymptote A2 were set to 4.2∙10^−5^ (GSC of IgG, data taken from Norden et al. [64]) and 1 (equaling complete sieving), respectively. Curve fitting gave the center parameter *LOGx*0 = 19.36833 and the Hill slope *p* = −0.12108. GSC_calc_ values were calculated via implementation of fitting parameters and the experimentally determined MW_app,norm._ in Equation (6).

## 4. Conclusions

In conclusion, AMSEC might offer the unique possibility to gain insight into human blood clearance kinetics, a fundamental aspect in the pharmacokinetics of radioligands already in vitro at the preclinical stage. If clinical data confirm this novel preclinical approach, AMSEC could furthermore contribute to continually ameliorated animal protection, as preclinical in vivo experiments (e.g., in rodents, with anyway limited transferability to humans) could be reduced to pre-selected compounds that exhibit favorable MW_app,norm._ and GSC_calc_ values. Thus, the implementation of AMSEC into the preclinical development process could help to further refine the identification of therapeutic lead compounds for clinical translation with suitable blood clearance kinetics in patients, hopefully fostering the development of next-generation (PSMA) radioligands for RLT with improved therapeutic efficacy.

## Figures and Tables

**Table 1:** Retention times of rhPSMA radioligands, PSMA-617, and PSMA-I&T in AMSEC runs (t*_R,AMSEC_*) and corresponding raw apparent molecular weights (MW_app,raw_).

Radioligand	t*_R,AMSEC_* ^1^ (min)	MW_app,raw_ (kDa) ^2^
rhPSMA-7.1	15.240	26.5
rhPSMA-7.2	14.564	32.0
rhPSMA-7.3	14.722 ^3^	30.6 ^3^
rhPSMA-7.4	14.143	36.0
rhPSMA10.1	15.410	25.3
rhPSMA-10.2	15.908	22.0
PSMA-617	17.586	13.8
PSMA-I&T	20.983	5.4

^1^ Retention times are corrected for the offset between UV-vis and radio-detector and are normalized to the elution behavior of the respective compound on gel filtration column 1 (for detailed information, see Equations (S1) and (S2) and Table S1 in the Supplementary Materials). ^2^ MW_app,raw_ corresponds to apparent molecular weights previously reported by *Wurzer* et al. [62]. Minor discrepancy in reported values (<1%) results from refined calibration calculations and correction for the offset between UV-vis and radio-detector in the present study (see Section 3.3). ^3^ Data presented for rhPSMA-7.3 are taken from an exemplary experiment. The mean MW_app,raw_ for rhPSMA-7.3 was 30.6 ± 0.5 kDa (n = 14).

**Table 2:** Retention factors *k*, normalized retention times (t*_R,norm._*), normalized apparent molecular weights (MW_app,norm._), and calculated glomerular sieving coefficients (GSC_calc_) of rhPSMA radioligands, PSMA-617, PSMA-I&T, and model compounds (MCs) 1 to 10.

Radioligand	Retention Factor *k*	t*_R,norm._* (min)	MW_app,norm._ (kDa)	GSC_calc_
rhPSMA-7.1	0.613	16.824	17.1	0.655
rhPSMA-7.2	0.680	15.962	21.7	0.344
rhPSMA-7.3	0.667 ^1^	16.125 ^1^	20.7 ^1^	0.408
rhPSMA-7.4	0.729	15.316	26.0	0.138
rhPSMA10.1	0.616	16.789	17.2	0.644
rhPSMA-10.2	0.543	17.736	13.2	0.846
PSMA-617	0.447	18.988	9.4	0.942
PSMA-I&T	0.001	24.792	1.9	0.992
MC-1	0.848	13.773	39.9	0.003
MC-2	0.798	14.414	33.3	0.020
MC-3	0.764	14.862	29.4	0.057
MC-4	0.747	15.081	27.7	0.089
MC-5	0.747	15.083	27.7	0.090
MC-6	0.658	16.248	20.0	0.454
MC-7	0.593	17.086	15.9	0.726
MC-8	0.540	17.781	13.1	0.852
MC-9	0.424	19.293	8.6	0.953
MC-10	0.194	22.287	3.7	0.987

^1^ Presented data for rhPSMA-7.3 were taken from an exemplary experiment. Mean retention factor *k* was 0.667 ± 0.06 (n = 14), and mean MW_app,norm._ was 20.7 ± 0.5 kDa (n = 14).

## Data Availability

Data is contained within the article and Supplementary Material.

## Supplementary Materials

The following supporting information can be downloaded at: https://www.mdpi.com/article/10.3390/ph15091161/s1, Table S1: Experimental retention times of AMSEC runs (t_R,AMSEC_ raw) and blank runs (t_R,blank_ raw), as well as corrected retention times converted to column 1 (t_R,AMSEC_ C1 and t_R,blank_ C1) of HSA, acetone, and all radioligands. Retention times of radiosignals are additionally corrected for the offset between UV-vis detector and radio-detector (t_R,AMSEC_ C1 + OC and t_R,blank_ C1 + OC). C1: column 1; OC: offset-corrected; n.a.: not applicable; Table S2: Retention times of model compounds (MCs) 1 to 10 in AMSEC runs (t*_R,AMSEC_*) and corresponding raw apparent molecular weights (MW_app,raw_); Table S3: Retention times of Blue Dextran 2000 (BD) and the calibration proteins conalbumin (CO, 75 kDa), ovalbumin (OV, 44 kDa), carbonic anhydrase (CA, 29 kDa), ribonuclease (RN, 13.7 kDa), and aprotinin (AP, 6.5 kDa) on superdex 75 increase columns 1, 2, and 3 (PBS pH 7.4 as mobile phase). *a* and *b*: calibration parameters; Cn: column n; V_e_: elution volume; V_0_: column void volume; R^2^: coefficient of determination of column calibration; Figure S1: Chemical structure of ^177^Lu-labeled rhPSMA-7.1 (*(R)*-configurated diaminopropionic acid branching unit and *(R)*-configurated DOTAGA chelator; Figure S2: Chemical structure of ^177^Lu-labeled rhPSMA-7.2 (*(S)*-configurated diaminopropionic acid branching unit and *(R)*-configurated DOTAGA chelator); Figure S3: Chemical structure of ^177^Lu-labeled rhPSMA-7.3 (*(R)*-configurated diaminopropionic acid branching unit and *(S)*-configurated DOTAGA chelator); Figure S4: Chemical structure of ^177^Lu-labeled rhPSMA-7.4 (*(S)*-configurated diaminopropionic acid branching unit and *(S)*-configurated DOTAGA chelator); Figure S5: Chemical structure of ^177^Lu-labeled rhPSMA-10.1 (*(R)*-configurated diaminopropionic acid branching unit and DOTA chelator); Figure S6: Chemical structure of ^177^Lu-labeled rhPSMA-10.2 (*(S)*-configurated diaminopropionic acid branching unit and DOTA chelator); Figure S7: Chemical structure of ^177^Lu-labeled PSMA-617; Figure S8: Chemical structure of ^177^Lu-labeled PSMA-I&T.
