# Supplementary material for: Albumin-Mediated Size Exclusion Chromatography: The Apparent Molecular Weight of PSMA Radioligands as Novel Parameter to Estimate Their Blood Clearance Kinetics
Source: Pharmaceuticals (Basel). 2022 Sep 19;15(9):1161. doi: 10.3390/ph15091161 (PMC9500755; doi:10.3390/ph15091161)
Supplement: Supplementary file 1 [file pharmaceuticals-15-01161-s001.zip › pharmaceuticals-1887113-supplementary.pdf]

Supplementary Material

Albumin-Mediated Size Exclusion Chromatography: The Apparent Molecular Weight of PSMA Radioligands as Novel Parameter to Estimate Their Blood Clearance Kinetics

Jan-Philip Kunert [1,*], Sebastian Fischer [1], Alexander Wurzer [1] and Hans-Jürgen Wester [1]

1 Technical University of Munich (TUM), Germany; TUM Department of Chemistry, Chair of Pharmaceutical Radiochemistry
* Correspondence: jan-philip.kunert@tum.de; Tel.: +49.89.289.12203

Calculation of corrected retention times and exemplary calculation of normalized apparent molecular weight ($MW_{app,norm.}$)

Throughout the development of the AMSEC method, three gel filtration columns of the same type (Superdex 75 Increase 10/300 GL gel filtration size exclusion column, fractionation range 70 – 3 kDa, GE Healthcare, Uppsala, Sweden) were used. The different columns gave slightly different retention times for the same probes, as exemplified by one of the calibration proteins, carbonic anhydrase (MW: 29 kDa), which showed retention times of 14.914 min, 14.352 min and 14.500 min, respectively. Calculations for the normalization of raw apparent molecular weights ($MW_{app,raw}$) were performed with the input of absolute retention times. Data acquired on different columns were combined in normalization calculations. Therefore, experimentally determined retention times of chromatographic runs performed on column 2 (three AMSEC-runs of rhPSMA-7.3) and column 3 (one AMSEC-runs of rhPSMA-7.3 and blank-runs) had to be converted to equivalent retention times on column 1 by means of the calibration parameters of the three columns, as described in the following.

Due to the calibration of each column, the calculated MW of the same probe was assumed to be identical when determined on two columns (see Equation S1). Equating the calculation of the probe's MW on e.g. column 1 (C1; calibration parameters a_{C1}, b_{C1} and $V_{0,C1}$) and column n (e.g. 2 and 3; Cn; calibration parameters a_{Cn}, b_{Cn}, and $V_{0,Cn}$), gives the following correlation (the formula for the calculation of MW is explained in the context of Table S3, see below):

$$MW = e^{\frac{\frac{0.8 \cdot t_{R,C1} - V_{0,C1}}{24 - V_{0,C1}} - b_{C1}}{a_{C1}}} = e^{\frac{\frac{0.8 \cdot t_{R,Cn} - V_{0,Cn}}{24 - V_{0,Cn}} - b_{Cn}}{a_{Cn}}} \qquad \text{(S1)}$$

The transformation of Equation S1 gives the retention time of column 1 ($t_{R,C1}$) as follows

$$t_{R,C1} = 1.25 \cdot \left\{ \left[\left(\frac{a_{C1}}{a_{C2}} \cdot \left(\frac{0.8 \cdot t_{R,C2} - V_{0,C2}}{24 - V_{0,C2}} - b_{C2} \right) \right) + b_{C1} \right] \cdot \left(24 - V_{0,C1}\right) + V_{0,C1} \right\} \qquad \text{(S2)}$$

Thus for a probe analyzed on column 2, an equivalent retention time on column 1 ($t_{R,C1}$) can be calculated with Equation S2 using no other input than the experimentally determined retention time on column 2 ($t_{R,C2}$) and the calibration parameters a, b and V_0 of both columns.

Experimentally detected retention times as well as retention times obtained from stepwise corrections for all the compounds are summarized in Table S1.

As an example, the calculation of the $MW_{app,norm.}$ of rhPSMA-7.1 was carried out as follows:

$$MW_{app} = e^{\frac{\frac{0.8 \cdot t_{R,norm.} - V_{0,C1}}{24 - V_{0,C1}} - b_{C1}}{a_{C1}}} = e^{\frac{\frac{0.8 \cdot 16.824 - 8.02880}{24 - 8.02880} - 2.09685}{-0.18028}} = 17067\ [Da] \tag{S3}$$

Table S1. Experimental retention times of AMSEC-runs ($t_{R,AMSEC}$ raw) and blank-runs ($t_{R,blank}$ raw) as well as corrected retention times converted to column 1 ($t_{R,AMSEC}$ C1 and $t_{R,blank}$ C1) of HSA, acetone and all radioligands. Retention times of radiosignals are additionally corrected for the offset between UV-vis-detector and radio-detector ($t_{R,AMSEC}$ C1 + OC and $t_{R,blank}$ C1 + OC). C1: column 1; OC: offset-corrected; n.a.: not applicable.

compound	column AMSEC	$t_{R,AMSEC}$ (min) raw	$t_{R,AMSEC}$ (min) C1	$t_{R,AMSEC}$ (min) C1 + OC[1]	column blank	$t_{R,blank}$ (min) raw	$t_{R,blank}$ (min) C1	$t_{R,blank}$ (min) C1 + OC
HSA	-	-	-	-	1	11.791	11.791	n.a.
acetone	-	-	-	-	3	24.596	24.809	n.a.
rhPSMA-7.1	1	15.324	15.324	15.240	3	20.509	20.795	20.711
rhPSMA-7.2	1	14.648	14.648	14.564	3	20.238	20.529	20.445
rhPSMA-7.3[2]	1	14.806	14.806	14.722	3	20.391	20.679	20.595
rhPSMA-7.4	1	14.227	14.227	14.143	3	20.271	20.561	20.477
rhPSMA-10.1	1	15.494	15.494	15.410	3	21.025	21.302	21.218
rhPSMA-10.2	1	15.992	15.992	15.908	3	20.605	20.889	20.805
PSMA-617	1	17.670	17.670	17.586	3	22.100	22.357	22.273
PSMA-I&T	1	21.067	21.067	20.983	3	20.798	21.079	20.995
MC-1	1	13.483	13.483	13.399	3	22.183	22.439	22.355
MC-2	1	14.170	14.170	14.086	3	23.022	23.263	23.179
MC-3	1	14.402	14.402	14.318	3	22.335	22.588	22.504
MC-4	1	14.607	14.607	14.523	3	22.433	22.684	22.600
MC-5	1	14.343	14.343	14.259	3	21.363	21.634	21.550
MC-6	1	15.096	15.096	15.012	3	21.006	21.283	21.199
MC-7	1	15.682	15.682	15.598	3	20.956	21.234	21.150
MC-8	1	16.408	16.408	16.324	3	21.458	21.727	21.643
MC-9	1	17.332	17.332	17.248	3	21.068	21.344	21.260
MC-10	1	19.151	19.151	19.067	3	20.615	20.899	20.815

[1] $t_{R,AMSEC}$ as given in Table 1 and Table S2. [2] Data presented for rhPSMA-7.3 are taken from an exemplary experiment.

Table S2. Retention times of model compounds (MC) 1 to 10 in AMSEC-runs ($t_{R,AMSEC}$) and corresponding raw apparent molecular weights ($MW_{app,raw}$).

radioligand	$t_{R,AMSEC}$ [1] (min)	$MW_{app,raw}$ (kDa)
MC-1	13.399	44.2
MC-2	14.086	36.5
MC-3	14.318	34.2
MC-4	14.523	32.4
MC-5	14.259	34.8
MC-6	15.012	28.2
MC-7	15.598	24.0
MC-8	16.324	19.6
MC-9	17.248	15.2
MC-10	19.067	9.2

[1] retention times are already corrected for the offset between UV-vis- and radio-detector and normalized to column 1 as described above.

Table S3. Retention times of Blue Dextran 2000 (BD) and the calibration proteins conalbumin (CO, 75 kDa), ovalbumin (OV, 44 kDa), carbonic anhydrase (CA, 29 kDa), ribonuclease (RN, 13.7 kDa) and aprotinin (AP, 6.5 kDa) on superdex 75 increase columns 1, 2, and 3 (PBS pH 7.4 as mobile phase). *a* and *b*: calibration parameters; Cn: column n; V_e: elution volume; V_0: column void volume; R^2: coefficient of determination of column calibration.

parameter	column 1	column 2	column 3
t_R(BD) (min)	10.036	9.657	9.676
t_R(CO) (min)	11.861	11.337	11.431
t_R(OV) (min)	13.036	12.491	12.607
t_R(CA) (min)	14.914	14.352	14.500
t_R(RN) (min)	17.399	16.891	17.031
t_R(AP) (min)	20.533	20.091	20.264
$V_{0,Cn}$ (mL)	8.0288	7.7256	7.7408
V_e(CO) (mL)	9.4888	9.0696	9.1448
V_e(OV) (mL)	10.4288	9.9928	10.0856
V_e(CA) (mL)	11.9312	11.4816	11.6000
V_e(RN) (mL)	13.9192	13.5128	13.6248
V_e(AP) (mL)	16.4264	16.0728	16.2112
a_{Cn}	−0.18028	−0.17873	−0.18032
b_{Cn}	2.09685	2.06926	2.09130
R^2_{Cn} (%)	99.2	99.1	99.1

Calibration parameters were determined as recommended by the manufacturer of Gel Filtration LMW Calibration Kits (GE Healthcare, Buckinghamshire, United Kingdom). In brief, the partition coefficient K_{av} was defined as:

$$K_{av} = \frac{V_e - V_0}{V_C - V_0} \quad \text{(S4)}$$

where V_e is the elution volume, V_0 is the column void volume, and V_C is the geometric column volume of 24 mL. K_{av} is semi-logarithmically plotted against MW, and the resulting linear calibration curve gives the calibration parameters *a* and *b* according to the following equation:

$$K_{av} = a \cdot ln(MW) + b \,. \tag{S5}$$

According to Equation S5, MW can be calculated from K_{av} and the calibration parameters *a* and *b* (Equation S6). K_{av} can be described according to Equation S4 wherein V_e is defined as the product of solvent flow (0.8 mL/min) with the retention time t_R resulting in Equation S6:

$$MW = e^{\frac{K_{av}-b}{a}} = e^{\frac{\frac{V_e - V_0}{V_C - V_0} - b}{a}} = e^{\frac{\frac{0.8 \cdot t_R - V_0}{24 - V_0} - b}{a}} \,. \tag{S6}$$

Chemical structures of ^{177}Lu-labeled rhPSMA compounds and ^{177}Lu-labeled references PSMA-617 and PSMA-I&T

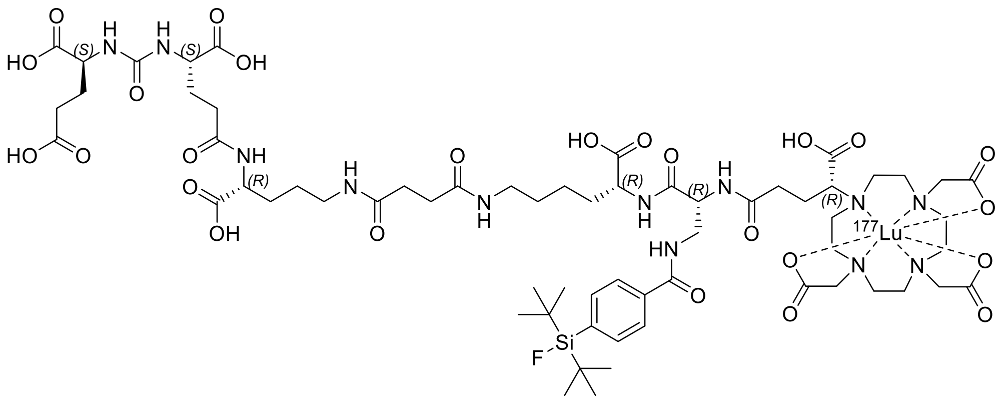

Chemical Formula: $C_{63}H_{96}F^{177}LuN_{12}O_{25}Si$
Molecular Weight: 1645,55

Figure S1: Chemical structure of ^{177}Lu-labeled rhPSMA-7.1 ((*R*)-configurated diaminopropionic acid branching unit and (*R*)-configurated DOTAGA chelator).

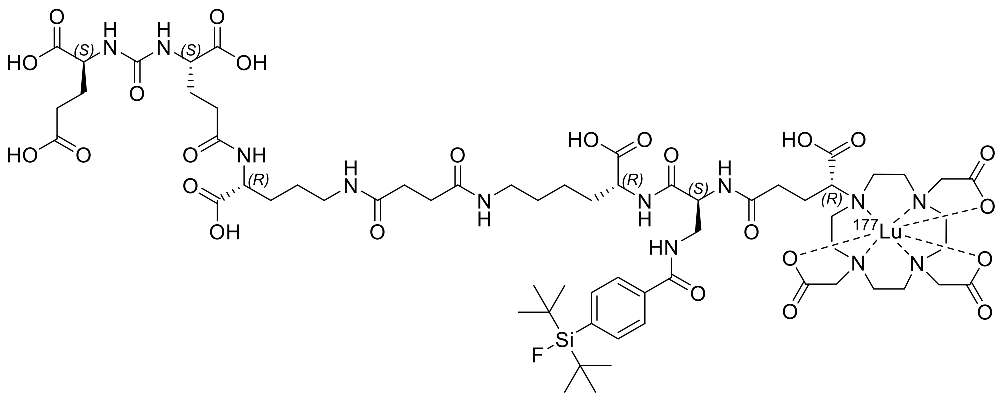

Chemical Formula: $C_{63}H_{96}F^{177}LuN_{12}O_{25}Si$
Molecular Weight: 1645,55

Figure S2: Chemical structure of ^{177}Lu-labeled rhPSMA-7.2 ((*S*)-configurated diaminopropionic acid branching unit and (*R*)-configurated DOTAGA chelator).

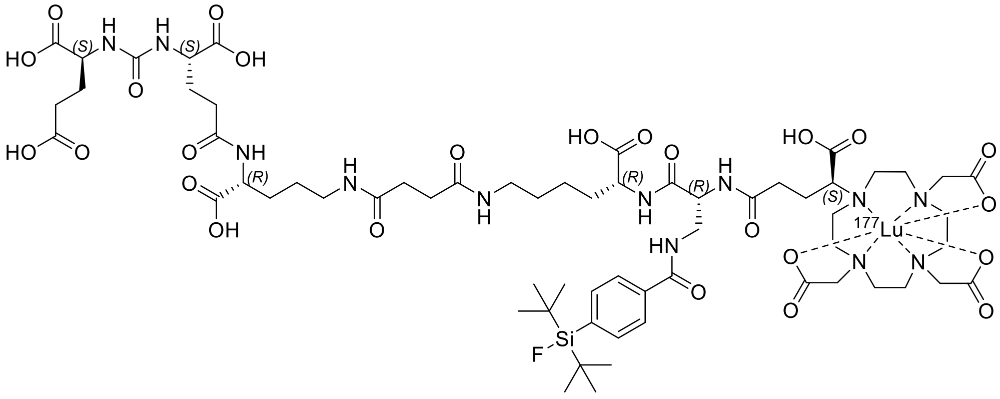

Chemical Formula: $C_{63}H_{96}F^{177}LuN_{12}O_{25}Si$
Molecular Weight: 1645,55

Figure S3: Chemical structure of ^{177}Lu-labeled rhPSMA-7.3 (*(R)*-configurated diaminopropionic acid branching unit and *(S)*-configurated DOTAGA chelator).

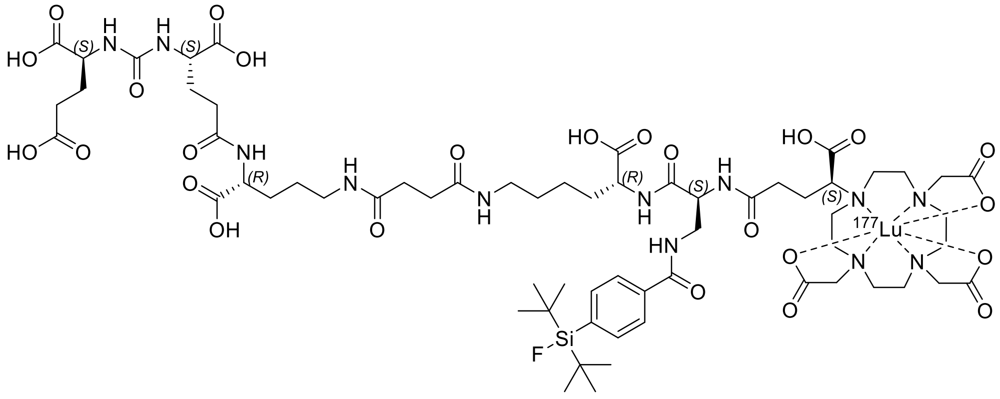

Chemical Formula: $C_{63}H_{96}F^{177}LuN_{12}O_{25}Si$
Molecular Weight: 1645,55

Figure S4: Chemical structure of ^{177}Lu-labeled rhPSMA-7.4 (*(S)*-configurated diaminopropionic acid branching unit and *(S)*-configurated DOTAGA chelator).

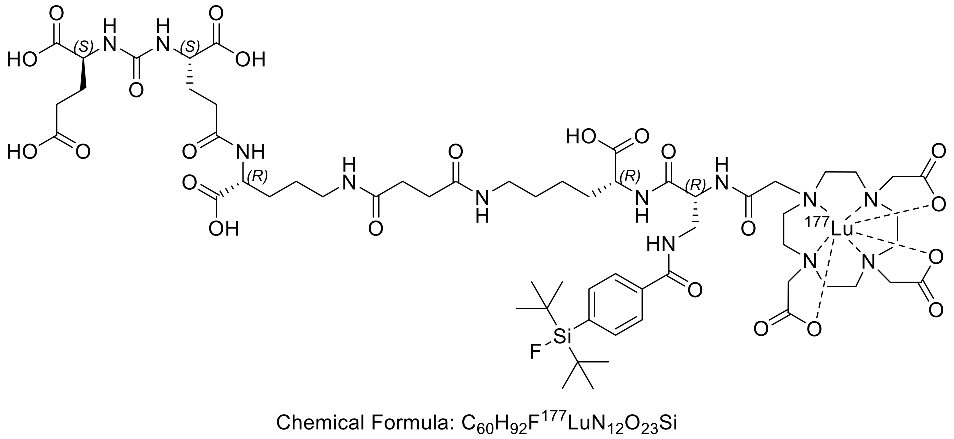

Chemical Formula: $C_{60}H_{92}F^{177}LuN_{12}O_{23}Si$
Molecular Weight: 1573,48

Figure S5: Chemical structure of ^{177}Lu-labeled rhPSMA-10.1 ((*R*)-configurated diaminopropionic acid branching unit and DOTA chelator).

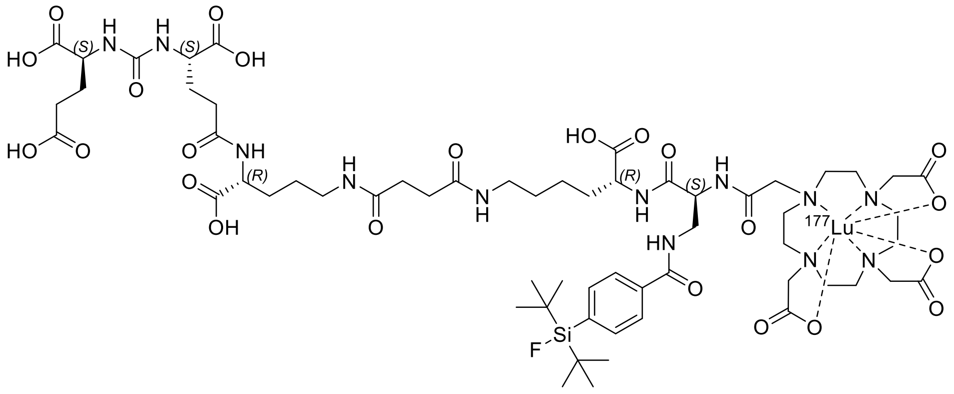

Chemical Formula: $C_{60}H_{92}F^{177}LuN_{12}O_{23}Si$
Molecular Weight: 1573,48

Figure S6: Chemical structure of ^{177}Lu-labeled rhPSMA-10.2 ((*S*)-configurated diaminopropionic acid branching unit and DOTA chelator).

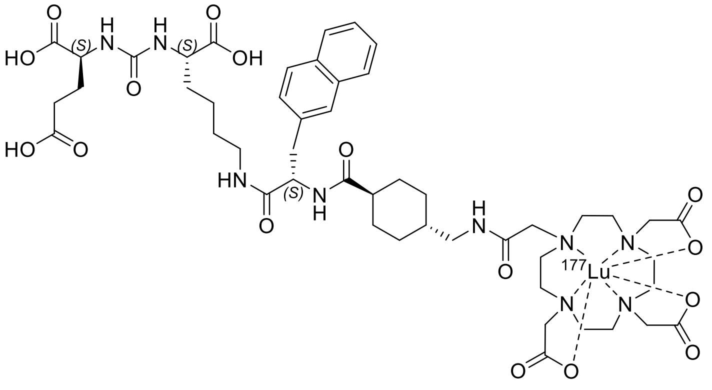

Chemical Formula: $C_{49}H_{68}{}^{177}LuN_{9}O_{16}$
Molecular Weight: 1216,07

Figure S7: Chemical structure of ^{177}Lu-labeled PSMA-617.

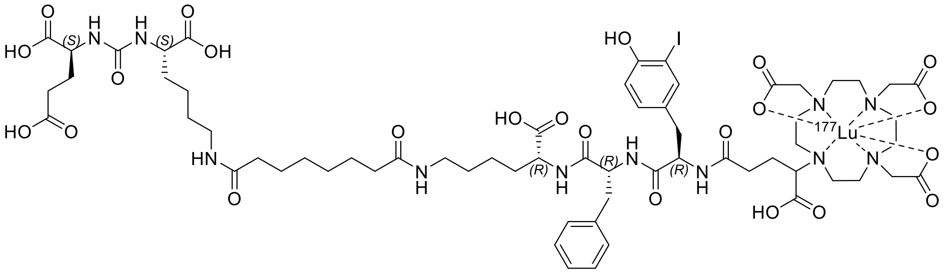

Chemical Formula: $C_{63}H_{89}I^{177}LuN_{11}O_{23}$
Molecular Weight: 1672,31

Figure S8: Chemical structure of ^{177}Lu-labeled PSMA-I&T.

Chemical structures of MC1-10 have not yet been disclosed.
